# Characterizing cardiac autonomic dynamics of fear learning in humans

**DOI:** 10.1111/psyp.14122

**Published:** 2022-06-07

**Authors:** Simone Battaglia, Stefano Orsolini, Sara Borgomaneri, Riccardo Barbieri, Stefano Diciotti, Giuseppe di Pellegrino

**Affiliations:** ^1^ Department of Psychology, Centre for Studies and Research in Cognitive Neuroscience University of Bologna Cesena Italy; ^2^ Department of Electrical, Electronic and Information Engineering University of Bologna Cesena Italy; ^3^ Department of Electronics, Information and Bioengineering Politecnico di Milano Milano Italy

**Keywords:** autonomic nervous system, fear conditioning, heart rate variability, psychophysiological responses, vagal control

## Abstract

Understanding transient dynamics of the autonomic nervous system during fear learning remains a critical step to translate basic research into treatment of fear‐related disorders. In humans, it has been demonstrated that fear learning typically elicits transient heart rate deceleration. However, classical analyses of heart rate variability (HRV) fail to disentangle the contribution of parasympathetic and sympathetic systems, and crucially, they are not able to capture phasic changes during fear learning. Here, to gain deeper insight into the physiological underpinnings of fear learning, a novel frequency‐domain analysis of heart rate was performed using a short‐time Fourier transform, and instantaneous spectral estimates extracted from a point‐process modeling algorithm. We tested whether spectral transient components of HRV, used as a noninvasive probe of sympathetic and parasympathetic mechanisms, can dissociate between fear conditioned and neutral stimuli. We found that learned fear elicited a transient heart rate deceleration in anticipation of noxious stimuli. Crucially, results revealed a significant increase in spectral power in the high frequency band when facing the conditioned stimulus, indicating increased parasympathetic (vagal) activity, which distinguished conditioned and neutral stimuli during fear learning. Our findings provide a proximal measure of the involvement of cardiac vagal dynamics into the psychophysiology of fear learning and extinction, thus offering new insights for the characterization of fear in mental health and illness.

## Abstract 2

Understanding transient dynamics of the autonomic nervous system during fear learning remains a critical step to translate basic research into treatment of fear‐related disorders. Here, using a novel frequency‐based analysis to capture transient changes of heart rate variability, our findings highlight a selective modulation of the high frequency (HF) band of the power spectrum, when facing fear‐relevant stimuli. Crucially, these results reveal specific increased parasympathetic (vagal) activity during fear learning in humans.

## 1 INTRODUCTION

Learning to respond to stimuli or circumstances that predict impending danger is a highly adaptive function for animals and humans alike. From an evolutionary perspective, learned fear serves to activate defensive responses in anticipation of harm, thus minimizing the impact of noxious challenge (Ploghaus et al., 2003). In the laboratory, a paradigm most often used to study this process is Pavlovian fear conditioning, wherein an initially neutral stimulus (the conditioned stimulus or CS), is paired with a noxious stimulus (usually a mild electric shock, the unconditioned stimulus, or US). As a result, after several pairings of the CS‐US, the presentation of the CS itself leads to a fear response (the conditioned response, or CR).

In humans, fear conditioning is often probed by measuring the activity of the autonomic nervous system (ANS), for instance via skin conductance responses (SCR) (Critchley et al., 2000), or pupil size responses (Kluge et al., 2011; Korn et al., 2017), or by assessing the fear‐potentiated startle (Brown et al., 1951; Khemka et al., 2017). Moreover, measures of heart rate variability (HRV), a noninvasive marker of autonomic control, are increasingly being employed, since the combination of several psychophysiological techniques may provide more precise quantification of the relationship between psychological and physiological processes in fear conditioning (Castegnetti et al., 2016; Paulus et al., 2016).

HRV refers to fluctuations of the length of time between consecutive heartbeats, or inter‐beat intervals. The heartbeat is generated by the sinoatrial node of the heart, and its occurrence is antagonistically modulated by the sympathetic and parasympathetic (vagal) branches of the ANS. Moreover, higher neural networks can exert a flexible control over the ANS, evoking reciprocal (i.e., increase in activity of one branch is associated with decreased activity in the other), or independent changes of the sympathetic and parasympathetic nervous systems (Berntson et al., 1991, 1993, 1994, 1996; de Geus et al., 2019; Koizumi & Kollai, 1992; Tessa et al., 2019).

Fear conditioned stimulus (CS) typically elicits transient heart rate deceleration (Hugdahl, 1995; Obrist et al., 1965) that reaches its nadir around the time of the US administration (Castegnetti et al., 2016; Tzovara et al., 2018). This anticipatory fear of bradycardia is generally believed to reflect almost exclusive vagal control. However, given the antagonistic effects of the sympathetic and parasympathetic branches, a bradycardic response to a stimulus could arise, for example, from either increased vagal activation, or sympathetic withdrawal, or even from vagal and sympathetic coactivation, in which the vagal effects exceed those due to the sympathetic nervous system (Jennings et al., 2016; Schipper et al., 2019). Therefore, simply tracking heart rate per se, may not provide an accurate rendition of these underlying autonomic mechanisms. A more accurate source of information is given by heart rate variations over time (i.e., HRV).

To gain deeper insight into the physiological underpinnings of fear learning, HRV has been employed here as a quantitative index of the interplay between sympathetic and parasympathetic influences on cardiac activity. Owing to the difference in their latencies of action (i.e., vagal effects unfold faster than sympathetic effects), the periodic oscillations in heart rate produced by the two autonomic branches occur at different frequencies (Akselrod et al., 1981; Chan et al., 2001). This serves as the basis for frequency‐domain techniques, such as spectral analysis, to distinguish between the frequency‐specific contribution of the sympathetic and parasympathetic systems to HRV at any given time. Spectral analysis allows the intensity of the HRV spectral components [i.e., the high‐frequency band (HF), low‐frequency band (LF), and very low‐frequency band (VLF)] to be determined. The HF component is believed to be mediated primarily by cardiac parasympathetic outflows and thus may provide a direct index of vagal activity; whereas the LF is commonly viewed as a product of both sympathetic and parasympathetic activity (Gianaros et al., 2004; Jennings & McKnight, 1994; Malliani et al., 1991; Nunan et al., 2010; Pumprla et al., 2002; Task Force, 1996).

Conventional analysis of HRV requires an observation window in the range of 2–3 minutes and some level of stationarity during this period (Akselrod et al., 1981; Chan et al., 2001; Rajendra Acharya et al., 2006; Task Force, 1996). It is, therefore, inadequate to capture transient changes in heart rate produced by the ANS, as those occurring over the duration of the CS presentation in a fear learning paradigm (typically a few seconds). In the present study, to investigate transient changes in response to fear learning, we used two novel approaches performing a frequency‐domain analysis of heart rate: short‐time Fourier transform (STFT) (for a more detailed explanation of the method, see [Oppenheim et al., 1999]), and instantaneous spectral estimates extracted from a “point‐process” modeling algorithm (for a more detailed explanation of the method, see [Barbieri et al., 2005]). The STFT method was used to examine the time‐frequency structure of the expected modulation of the autonomic regulation. Because this method is known to introduce a spectral spread of the components, a point‐process modeling algorithm was used to obtain unbiased time‐varying spectral estimates. Indeed, the point‐process allows to compute instantaneous estimate of HRV based on a probabilistic generative mechanism of the heartbeat at each moment in time. It is therefore particularly effective to finely track changes in the temporal dynamics of heartbeat intervals.

In this study, we tested whether the spectral components of the HRV, as a noninvasive marker of sympathetic and parasympathetic mechanisms, can dissociate between conditioned and neutral stimuli related to fear learning. To this end, we combined the electrocardiogram (ECG) signal recording with an established psychophysiological measure of fear conditioning that is the SCR. The results of the present study should thus provide unique insights into the psychophysiological metrics of fear learning, widening our understanding of patients suffering from several psychiatric conditions (i.e., anxiety‐related disorders and PTSD) (Borgomaneri et al., 2020; Borgomaneri, Battaglia, Avenanti, & di Pellegrino, 2021; Borgomaneri, Battaglia, Sciamanna, et al., 2021; Herrmann et al., 2017).

## 2 METHODS

### 2.1 Subjects

A total of 54 healthy individuals were enrolled in the present study; however, due to technical problems a total of 50 individuals took part in the experiment (29 female; mean age ± *SD* = 24.02 ± 2.99 years; mean education ± *SD* = 14.98 ± 2.06 years). Prospective participants were recruited from the student population of the University of Bologna using campus advertisements. All subjects were right‐handed as assessed by the Edinburgh Handedness Inventory (Oldfield, 1971), had normal or corrected‐to‐normal visual acuity in both eyes, and were naive to the purposes of the experiment. Individuals who reported a history of psychiatric care, neurological disease, cardiovascular conditions, or substance abuse were excluded, as were individuals currently treated with any medication known to affect the central nervous system. Trait anxiety and depression were measured, given evidence for their relationship with fear learning (Otto et al., 2007; Prenoveau et al., 2011). State anxiety was assessed using the State–Trait Anxiety Inventory (Spielberger et al., 1983), which possesses good reliability and validity. Depression symptomatology was assessed with the Hospital Anxiety and Depression Scale (Zigmond & Snaith, 1983), which has moderate to high convergent validity. However, none of the participants showed a level of pathological anxiety (mean ± *SD* = 39.24 ± 6.51) or depression (mean ± *SD* = 3.18 ± 1.97) (Emons et al., 2019). All participants gave informed written consent to participate after being informed about the procedures of the study. All procedures were conducted in accordance with the ethical principles of the World Medical Association Declaration of Helsinki and were approved by the Ethics Committee of the Department of Psychology of the University of Bologna.

### 2.2 Apparatus, stimuli, and task

The study was implemented in a MATLAB environment (version R2018b; The MathWorks, Inc., Natick, Massachusetts, USA) on a Windows‐based PC (Lenovo ThinkCentre Desktop Computer). A classical fear conditioning paradigm with partial reinforcement was used. The task consisted of habituation, Acquisition, and Extinction phases presented continuously. Unconditioned stimulus (US) was a 200‐ms train of electrical square pulses (individual pulse width of 0.2 ms), generated by a constant‐current stimulator (DS7A, Digitimer Ltd., UK), and applied via two surface electrodes fixated on the inner side of the participants' left wrist. The intensity of the electrical stimulus was determined individually by assessing the participants' subjective evaluation in a standard workup procedure before the experimental task. The current was initially set at 0.5 mA and increased in steps of 1 mA until participants reported it as a “highly annoying, but not painful” stimulation (participant's mean ± *SD* = 7.92 ± 2.32 mA). Conditioned stimuli (CSs) consisted of two visual stimuli created with Blender (Blender Foundation, Amsterdam, Netherlands), and Cinema 4D R17 software (MAXON Computer GmbH, Friedrichsdorf, Germany), and presented on a computer screen. CSs were images of two different indoor environments (i.e., a yellow‐blue room, and a gray‐red room) that covered the entire screen (adapted from previous studies [Battaglia et al., 2018, 2020; Borgomaneri et al., 2020]). Neutral, rather than intrinsically emotional (i.e., spiders, snakes, or angry faces), visual stimuli were used as CSs, because conditioned responses to very salient CSs can be confounded by the ceiling effects of the respective outcome measures (Bevins & Ayres, 1991; Lonsdorf et al., 2017). Finally, the type of stimuli associated with the CS+ and CS− were counterbalanced across participants.

Regarding the experimental paradigm, each trial consisted in the presentation of one CS in the center of a computer screen for 4 s, followed by an inter‐trial interval (ITI) of variable duration, from 14 to 17 s, during which the screen turned completely gray and empty. During the task, trials were pseudo‐randomly presented to participants such that no more than three identical CSs occurred in a row. The experiment starts with the habituation phase, during which the CSs were presented without reinforcement. Two habituation trials were used to avoid retardation of learning due to non‐reinforced exposure to CS+ (i.e., the latent inhibition effect [Reiss & Wagner, 1972]). Habituation trials were not analyzed. During the subsequent Acquisition phase (32/40 trials), one CS was designated as CS+ and was associated with the US 60% of the times, while the unreinforced stimulus (CS−) was a different visual stimulus not associated with any consequence (Marin et al., 2017; Milad et al., 2007). In CS+ trials, the US was administered 3.8 s after the CS+ onset and co‐terminated with the CS+, 0.2 s later. Finally, during the Extinction phase (32/40 trials), both CS+ and CS− stimuli were presented without the US administration.

### 2.3 Procedure

The study was performed at the Centre for Studies and Research in Cognitive Neuroscience of the University of Bologna, in Cesena, Italy. Participants were tested individually. They were comfortably seated in a silent and dimly lit room in front of a computer screen (size: 43 inches; resolution: 1920 × 1080 pixels; refresh rate: 60 Hz), at ~75 cm viewing distance. Once seated, the experimental procedure was explained, and written informed consent was obtained from participants. To ensure that SCR was correctly recorded, before testing each participant, the responsiveness of SCR to loud and sudden sounds was checked. Thus, after verifying that signals were being properly acquired by the instruments, and the intensity of the electrical stimulation was set, the experiment started. Participants were instructed that different images would be presented on the screen and that they would have to carefully observe the screen, as some of the displayed stimuli might be paired with electrical stimulation. During the experiment, visual and electrical stimuli were automatically administered by the task presentation system implemented in a MATLAB environment, while ECG and SCR signals were recorded continuously.

### 2.4 Physiological signal recordings

In the present study, signals were recorded with a Biopac MP‐150 system at 200 Hz sampling rate and fed into AcqKnowledge 3.9 software (BIOPAC Systems, Inc., Goleta, California, USA) for offline analysis. The SCR was acquired with two Ag/AgCl electrodes (TSD203 model; BIOPAC Systems) filled with isotonic hyposaturated conductant gel (GEL101 model; BIOPAC System), and attached to the distal phalanges of the second and third finger of the participant left hand. A Biopac EDA100C module was used to amplify the SCR signal (gain switch set to 5 μS/V, low pass to 35 Hz, high pass to DC). The ECG was acquired with three Ag/AgCl electrodes (EL503 model; BIOPAC Systems) filled with isotonic hyposaturated conductant gel (Lectron III Gel, NEUROSPEC). Electrodes were positioned in a modified bipolar lead I configuration, with the positive electrode placed on the participant's left wrist, the negative electrode on the right wrist, and the ground electrode attached just underneath the right clavicle. A Biopac ECG100C module was used to amplify ECG signals (gain switch set to 500, low pass to 35 Hz, high pass to 0.05 Hz).

### 2.5 SCR data processing and statistical analysis

SCR data were analyzed offline in a MATLAB environment, and all statistical analyses were performed with STATISTICA (StatSoft, v. 13.0, Round Rock, Texas, USA). SCR following the CS was analyzed to assess conditioned responses, whereas SCR following the US was analyzed to assess unconditioned responses. The onset was represented respectively by the time of stimulus presentation and electrical shock administration. Each trial was extracted from the entire SCR signal and the peak‐to‐peak value was calculated as the amplitude of the largest deflection during the 0.5 to 4.5 s time window after stimulus onset. The minimum response criterion was 0.02, and smaller responses were encoded as zero. Then, SCR peak‐to‐peak values were square‐root transformed and scaled to each participant's average square‐root of US responses (Battaglia et al., 2018, 2020; Garofalo et al., 2017) to reduce interindividual variability and increasing statistical power (Schiller et al., 2008; Siddle et al., 1988). Finally, SCR values were collapsed into “early” and “late” responses for each sub‐phase of Acquisition and Extinction, as learning typically varies across time (Grady et al., 2016; Lonsdorf et al., 2017). Learning‐related changes in SCR were hypothesized to be found in the “late” sub‐phase of both Acquisition and Extinction, as previously reported (Battaglia et al., 2018; Dunsmoor et al., 2018; Merz et al., 2014; Milad et al., 2005; Schiller et al., 2010). The normality of data distribution was verified with Shapiro–Wilk tests. Mixed‐design analyses of variance (ANOVAs) were used to investigate differences within experimental phases and Scheffè post hoc analyses were conducted. Finally, a bootstrap procedure with 1000 samples was used to recalculate our *p* values to estimate the precision of our statistics (see Kuhn et al., 2022; Wright et al., 2011).

### 2.6 Identification of QRS complex peaks

ECG processing and analyses were performed in a MATLAB environment. Identification of QRS complex peaks from the ECG was carried out automatically by an in‐house developed sample‐based envelope detector algorithm (see Supplementary Materials). Plots of subject's ECG and resulting inter‐beat intervals (IBI) sequence were presented to the operator and identified peaks appeared on the ECG plot to allow a quality check and interactive corrections (see Figure S1). A trained operator was instructed to inspect the resulting sequences, manually correct for misidentifications, and regularize singular ectopic events using linear interpolation (Nabil & Bereksi Reguig, 2015). Furthermore, the operator reconstructed the IBI sequence during the administration of US and reported that none of the enrolled participants presented multiple consecutive ectopic events.

### 2.7 Signal processing of heart rate dynamics

RR‐interval sequence was obtained by homogeneous resampling of the IBI sequence at 10 Hz (Singh et al., 2004) (see Supplementary Materials). The value associated with average heart rate was removed from the RR‐interval sequence subtracting the moving‐median computed over the past T seconds to preserve causality (i.e., the moving‐median does not depend on the future values of the signal); the median was chosen as a more robust estimator than the mean for skewed distributions and in presence of outliers (Hedges & Shah, 2003) (see Figure S2). The time window length T was chosen to be 15 s to encompass the entire duration of the RR‐interval response as observed under similar experimental conditions (Castegnetti et al., 2016). The STFT was computed using a centered Hamming window of length 15 s, at time periods of 0.10 s, with an interpolated spectral resolution of 0.01 Hz for components from 0 to 0.50 Hz. Finally, since specific changes in heart rate dynamics were hypothesized to be found in the late sub‐phase of both Acquisition and Extinction, analysis specifically investigated heart rate changes in the late phases (Battaglia et al., 2018; Dunsmoor et al., 2018; Merz et al., 2014; Milad et al., 2005; Schiller et al., 2010).

### 2.8 Statistical analysis of STFT and HRV indices

Each spectral component of the STFT, and the HRV indices, were normalized using a moving modified z score (for a more detailed explanation see [Iglewicz & Hoaglin, 1993]), where the median and the median of absolute distances (MAD) were calculated over the past T seconds to preserve causality. For each spectral component, the numerator of the moving modified z score plays the role of removing the uninformative trends (tonic component) allowing to focus the analysis on transitory oscillations (phasic component), while the denominator is used to scale for variability allowing for both inter‐component and inter‐subject comparability. Single trial responses of the RR‐interval, STFT, and HRV indices were considered in a time window spanning 3 s before and 15 s after the CS onset and analyzing only the late sub‐phase of both Acquisition and Extinction. Based on trials' collections, a nonparametric Mann–Whitney *U* test (TMW) was performed for each spectral component at each time point, to compare CS+/US against CS− and CS+ against CS−. The number of spectral components *N* were set at 50 for the STFT analysis and 4 for the HRV indices analysis. Since at each timepoint spectral power tends to appear in distinct clusters (i.e., if one spectral component is significant it is likely that adjacent components will be as well) we cannot assume independence of the statistics computed along the *N* spectral components. Nevertheless, such dependence resembles that of gene paths which comes in “relatively small, disjoint groups” (Storey, 2003), therefore, under this assumption, positive false discovery rate (pFDR) correction was computed at each time‐point. Convergence of pFDR is reliably achieved when computed on the order of 1000 tests (Storey, 2003), thus a comparable amount of spectral components should be estimated. However, such an approach would serve little practical purpose and imply a superfluous computational burden. Instead of constraining the number of spectral components *N* to the correction requirements, we exploited the generation of *B* bootstrap replicates of the TMW statistics to obtain *B* times *N p* values at each time point. The *B* resampling of data was created randomizing trials with replacement (pooling was not involved and collections numerosity was preserved). With this approach, once the number of spectral components *N* has been defined, the sufficient amount of *B* bootstrap replicates can be calculated as the number of tests divided by N. In this study, we opted to compute the pFDR on 2000 tests giving *B* = 40 for the STFT analysis and *B* = 500 for the HRV indices analysis. The resulting corrected *p* values were approximated by the median computed over the *B* values associated with each of the *N* spectral components (Bhattacharya & Habtzghi, 2002).

### 2.9 Point‐process modeling of heart rate dynamics

The sequence of systolic peaks timing was passed to an autoregressive (AR) point‐process modeling algorithm (Barbieri et al., 2005) to compute instantaneous estimates of heart rate variability defined in the time and frequency domains, with regression order *p* = 16, local likelihood interval *l* = 120 s, weighting coefficient *α* = .01, and updating interval Δ = 5 ms. All parameters were determined after preliminary goodness‐of‐fit analysis of the data with the evaluation of Kolmogorov–Smirnov statistic. This approach models the stochastic nature of heartbeat generation considering a physiologically plausible, history‐dependent, inverse‐Gaussian process of ventricular repolarization (Barbieri et al., 2005). This allowed us to obtain an instantaneous RR‐interval mean estimate at a very fine timescale, which required no interpolation between the arrival times of two beats. Moreover, we were able to use the instantaneous *p* coefficients of the AR model to compute the distribution of spectral powers (for a more detailed explanation, see [Mainardi, 2009]). HRV indices were computed integrating spectral powers within the four frequency ranges of interest (VLF, LF, HF_inf_, and HF_sup_) and down‐sampled at 10 Hz as a data compression solution without loss of relevant information.

## 3 RESULTS

We first ensured that participants successfully learned the contingency between stimuli and relative outcomes. To this end, we contrasted the changes in response to CS+ and CS− by means of SCR as an established psychophysiological measure of fear conditioning. Once fear Acquisition and Extinction were confirmed, our main goal was to characterize the autonomic signatures of heart rate modulation and to reveal specific spectral dynamics in response to fear conditioned stimuli.

### 3.1 SCR results

SCR data were analyzed using a 4 × 2 repeated measure ANOVA, with Phase (Acquisition early/Acquisition late/Extinction early/Extinction late) and Stimulus (CS+/CS−), as within‐subject factors. Results show a main effect of Phase (*F*(3,147) = 14.813, *p* < .001, *η* ^2^ = .23), which reflected changes in SCRs during the different phases of the experiment, and a main effect of Stimulus (*F*(1,49) = 51.617, *p* < .001, *η* ^2^ = .51) reflecting specific SCRs differential changes in response to the stimuli presented. Crucially, a significant Phase × Stimulus interaction (*F*(3,147) = 11.768, *p* < .001, *η* ^2^ = .19) was found. Follow‐up post hoc analysis showed a different pattern of SCRs in response to stimuli between phases. Specifically, during both early (CS+ mean ± *SD* = 0.54 ± 0.19 μS; CS− = 0.43 ± 0.20 μS), and late (CS+ = 0.50 ± 0.24 μS; CS− = 0.31 ± 0.16 μS) phase of Acquisition, SCR to CS+ was higher than to CS− (all *p* < .001), suggesting a successful fear learning. Moreover, bootstrap analysis revealed that the comparison between CS+ and CS− during Acquisition was found to be significant (*p* = .001), confirming that the acquisition of conditioning had occurred. Subsequently, during the early phase of extinction, the previous conditioned response to CS+ (mean ± *SD* = 0.48 ± 0.31 μS) was higher than to CS− (0.38 ± 0.23 μS; *p* < .001), due to the strength of the acquired conditioning and the few extinction trials presented. In opposition, in the late phase of Extinction, no difference in SCR was found between CS+ (0.30 ± 0.28 μS) and CS− (0.25 ± 0.21 μS; *p* = .25), indicating that extinction had occurred. Together SCR results demonstrate that participants showed stronger responses to CS+ than to CS− during Acquisition, which decreased until the difference between CS+ and CS− disappeared during late extinction. Overall, these findings indicate successful discriminative Acquisition of fear learning and subsequent Extinction.

### 3.2 ECG results

In the following sections, the *R* waves extracted from the ECG have been analyzed by contrasting the estimates dynamics between the stimuli presented, to characterize the autonomic modulation of heart rate during fear conditioning in both Acquisition and Extinction late phases.

#### 3.2.1 Heart period (RR‐interval) responses

**FIGURE 1:**
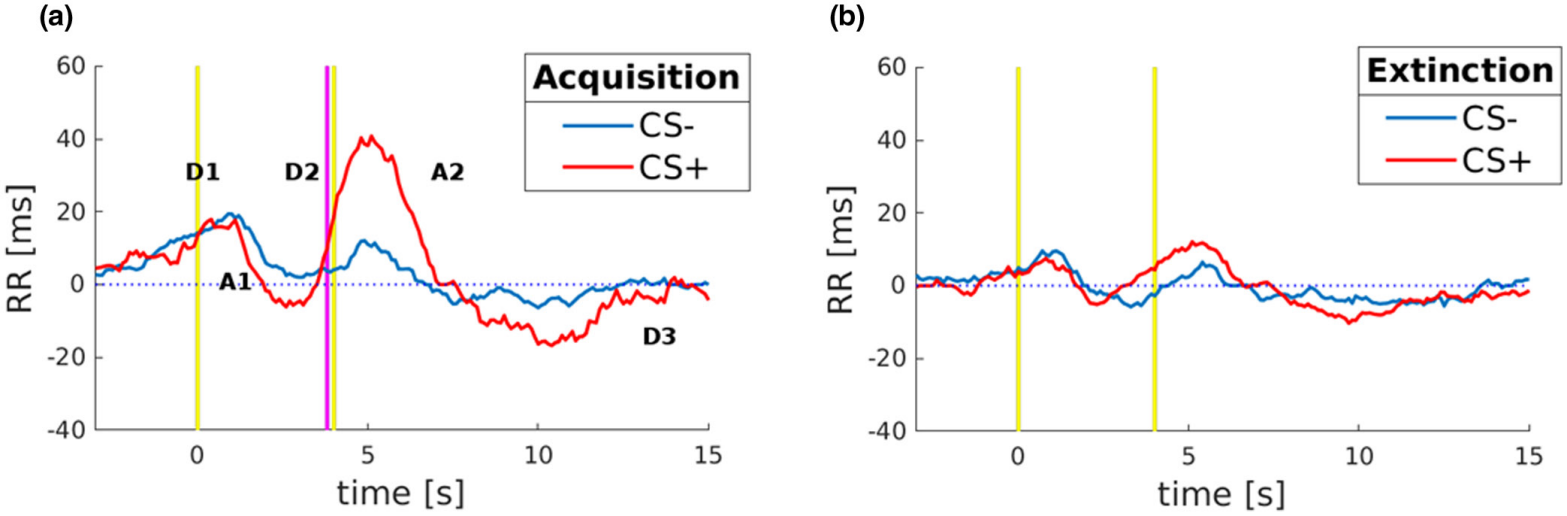
RR‐interval responses to stimuli. CS+ and CS− have been decomposed into their components of heart rate deceleration (positive slope of RR) and acceleration (negative slope of RR), respectively labeled as “D” and “A”. During Acquisition (a), RR‐interval to CS+ differs from CS−, reflecting prominent bradycardia (D2) when the shock administration is expected. Importantly CS+ responses are referred to those without the shock received. During the Extinction (b), RR‐interval to CS+ and CS− showed equivalence in heart rate modulation. In graphs, yellow vertical lines represent the onset and the offset of both the CS+ and CS−. The pink vertical line represents where the shock would occur after the CS+ presentation.

The time elapsed between heartbeats is usually referred to as “RR‐interval” and measured in milliseconds (ms). Thus, the heart period responses have been decomposed into their deceleration (positive slope of RR) and acceleration (negative slope of RR) components. The grand medians of the RR‐interval to CS+ and CS− are depicted in Figure 1. In line with previous studies of classical fear conditioning (Bohlin & Kjellberg, 1979; Castegnetti et al., 2016; Paulus et al., 2016), in response to CS+ presentation during the Acquisition, it was observed an initial deceleration (D1 slope, Figure 1a), usually considered to reflect a basic stimulus registration process (Barry, 1984), followed by an acceleration (A1 slope, Figure 1a), and a prominent continuing deceleration (D2 slope, Figure 1a). Also, to investigate how the cardiac signals change until their return to baseline level, we extended the time window and disclosed the observation of two more components: a second acceleration (A2 slope, Figure 1a) and a final deceleration to cardiac baseline activity (D3 slope, Figure 1a). Crucially, Figure 1a shows that the grand medians of response to CS+ differ from those to CS−, reflecting prominent bradycardia induced by the presentation of the CS+ (D2 positive slope) which continued approximately until 1 s after the expected time of US administration and then returned to baseline slowly (Figure 1a). Instead, during Extinction, dynamics in response to the CS+ and CS− showed equivalence in heart rate modulation, indicating that Extinction has indeed occurred (Figure 1b). Taken together these results revealed that during Acquisition, RR‐interval to CS+ showed qualitatively larger dynamics than CS−. On the other hand, during Extinction, RR‐interval to CS+, and CS− showed superimposed and nonselective signal in heart rate modulation.

#### 3.2.2 Frequency domain HRV analysis

**FIGURE 2:**
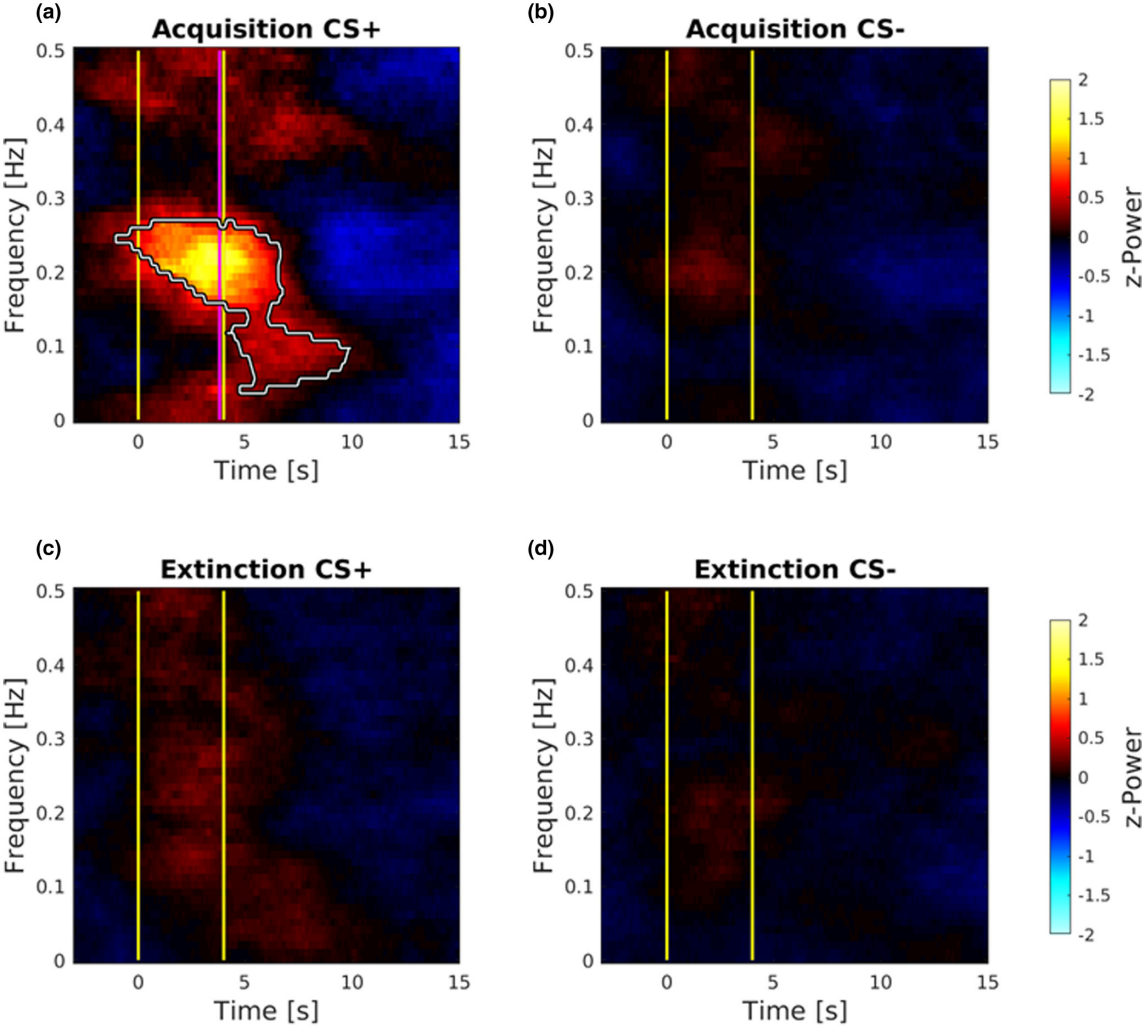
Normalized power of STFT responses to stimuli. Statistical differences are represented as the areas within white boundaries (*p* < .001). During Acquisition (a and b), the comparison between CS+ and CS− revealed a larger power contribution in the range of 0.05 to 0.30 Hz. Importantly, CS+ responses are referred to those without the shock received. During Extinction (c and d), no differences were found contrasting CS+ and CS− responses. In graphs, yellow vertical lines represent the onset and the offset of both the CS+ and CS−. The pink vertical line represents where the shock would occur after the CS+ presentation.

Responses of the normalized power (*z*‐Power) of STFT for CS+ and CS− in both Acquisition and Extinction are depicted in Figure 2, and statistically significant results (*p* < .001) were highlighted with a colored white boundary. In particular, during Acquisition, comparison analysis between CS+ against CS−, showed a significant cluster of power contribution from 0.05 to 0.30 Hz, with a higher concentration of power at 0.21 Hz, which is occurring at 3.90 s (Figure 2a), approximately the time in which participants expect the shock administration (for further statistical details see Table 1). During the Extinction, comparison analysis between CS+ and CS− showed no significant differences in power contribution after stimuli presentation (Figure 2c,d).

**TABLE 1:** STFT statistics

Stimulus	STFT median (*z*‐Power)	STFT MAD (*z*‐Power)	Time (s)	Frequency (Hz)	*p* Value
CS+	1.66	4.05	3.90	0.21	6.4 × 10^−12^
CS−	0.07	2.58			

*Note*: Time and frequency of STFT *z*‐Power maxima in the difference between CS+ and CS− responses were identified for the significant cluster (*p* < .001). STFT median and median of absolute distances (MAD) values of *z*‐Power are reported separately for each stimulus during Acquisition. The column *p* value contains the significance level of the comparison at the identified maximum difference.

**FIGURE 3:**
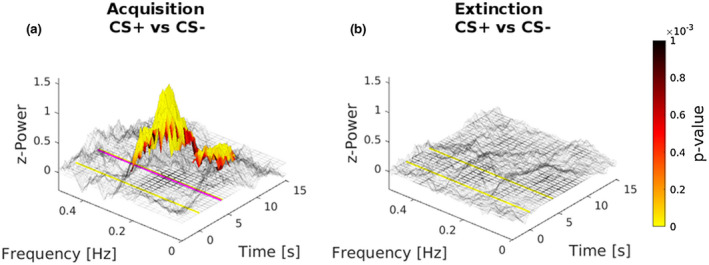
Maps of *p* value distribution. Maps were generated to evaluate the distribution of the significance level in the resulting significant cluster. Maps were superimposed over a gray‐scale mesh representing the difference of STFT *z*‐Power between CS+ and CS− responses in both the Acquisition (a) and Extinction (b) phase. Importantly, CS+ responses are referred to those without the shock received during Acquisition. In graphs, yellow vertical lines represent the onset and the offset of both the CS+ and CS−. The pink vertical line represents where the shock would occur after the CS+ presentation.

Importantly, maps of *p* value were also generated to evaluate the distribution of the significance level in the resulting significant cluster (*p* < .001; Figure 3). Thus, STFT *z*‐Power was superimposed over a gray‐scale mesh representing the difference of power contribution between the analyzed responses of CS+ against CS− in the Acquisition (Figure 3a), and in the Extinction phase (Figure 3b). Furthermore, for the significant cluster (with the maximum concentration of power at 0.21 Hz), maxima in the difference between CS+ and CS− responses were identified, STFT *z*‐Power values were extracted classifying times and frequencies, and significance level of the comparison are reported in Table 1.

Taken together these results indicate that the presentation of CS+, as compared to CS−, elicits a strong response in the frequency range of 0.15–0.30 Hz, sustaining specific vagal contribution, thus explained bradycardia induced in response to fear conditioned stimulus (Figures 2a,b and 3a; Table 1). Importantly, this implies that fear conditioned stimulus has been able to trigger vagal branches of ANS, slowing the heartbeats, as revealed by the spectral analysis showing a significant cluster within the frequency band of vagal contribution (Malliani et al., 1991; Pumprla et al., 2002; Task Force, 1996). Also, results indicate no difference in the spectral content of CS+ as compared to CS− during Extinction (Figures 2c,d and 3b). Crucially, these results revealed and quantified the selective vagal contribution as a response to fear conditioned stimulus, thus reflecting the mere parasympathetic activity in the anticipation of threat.

#### 3.2.3 Point‐process modeling of heart rate dynamics

**FIGURE 4:**
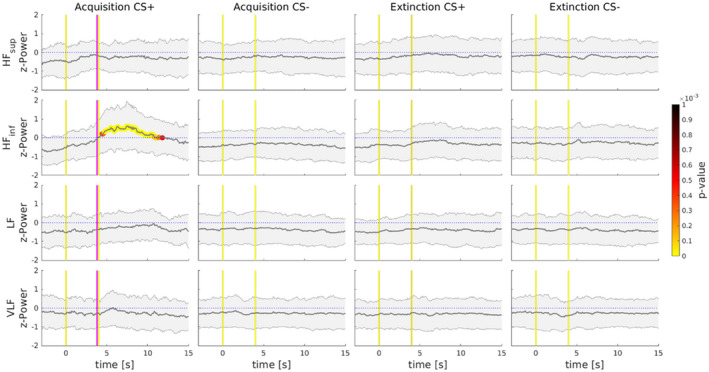
Point‐process modeling of heart rate dynamics. Graphs show normalized indices of median and median of absolute distances (MAD) responses for stimuli in both Acquisition and Extinction. During Acquisition, a significant increase of HF_inf_ is highlighted and colored signal represent the p‐value timepoints of significance (*p* < .001), in response to CS+ as compared to CS−. Importantly, CS+ responses are referred to those without the shock received. During Extinction, no significant differences in any range of frequencies investigated were found between stimuli. In graphs, yellow vertical lines represent the onset and the offset of both the CS+ and CS−. The pink vertical lines represent where the shock would occur after the CS+ presentation.

To further investigate unbiased spectral powers in light of the STFT results, a point‐process modeling algorithm of cardiac dynamics was adopted to extract instantaneous spectral power indices of heart rate. These were calculated in four frequency ranges: very low frequencies (VLF) [0.003 0.03) Hz, low frequencies (LF) [0.03 0.15) Hz, inferior range of high frequencies (HF_inf_) [0.15 0.30) Hz, and superior range of high frequencies (HF_sup_) [0.30 0.45) Hz. Stimuli responses of the normalized estimated indices by the point‐process modeling are depicted in Figure 4 and a statistical comparison analysis between CS+ against CS− was conducted separately for each experimental phase. During the Acquisition phase, responses to CS+, as compared to CS−, showed significantly increased contribution (*p* < .001) in the range of HF_inf_ around 4 s, and it was sustained until cardiac baseline was restored (Figure 4). No other significant differences were found in the other frequency ranges during Acquisition. Moreover, CS+ and CS− comparison during the Extinction phase showed no significant differences in any range of frequencies investigated (Figure 4). Finally, using a point‐process modeling algorithm, results confirm specific increases of the HF_inf_, associated with the vagus nerve involvement, as well as its temporal involvement in response to CS+ when the shock is expected to be administered.

## 4 DISCUSSION

In humans, fear conditioning is usually assessed by skin conductance response (SCR) (Critchley et al., 2000) and fear potentiated startle (FPS) (Brown et al., 1951). However, both SCR and FPS present some methodological and practical limitations. As a matter of fact, SCR is subject to a fast habituation decay, whereas FPS requires the presentation of US during both CS+ and CS− leading to possible interference in the learning process (Castegnetti et al., 2016). Although previous research has extensively used heart rate to assess sustained autonomic tone, recent studies have shown that it can also be used as a tool to investigate more phasic changes, such as conditioned responses in emotional learning (Castegnetti et al., 2016; Liu et al., 2013; Pappens et al., 2014; Paulus et al., 2016; Sevenster et al., 2015; Tzovara et al., 2018; Wendt et al., 2015). Specifically, it has been reported that fear conditioned stimuli (CS+) typically elicit transient heart rate deceleration, and this anticipatory fear bradycardia is generally believed to reflect vagal control. The use of diverse and complementary methodologies to study physiological responses of fear conditioning may contribute to the early identification of individuals prone to the development of psychiatric disorder (Sevenster et al., 2015). One crucial difference between the two measures is that, while SCR is under the almost exclusive control of the sympathetic nervous system (SNS) (Boucsein, 2012), the HRV may be under the control of the sympathetic as well as the parasympathetic nervous system (Berntson et al., 2007; Ernst, 2017). Thus, a measure able to disentangle the contribution of the two systems is highly desirable.

To achieve this goal, we analyzed SCR as a measure of the sympathetic nervous system activity and results showed that fear Acquisition and Extinction have been occurred, in particular demonstrating higher SCR to CS+ as compared to CS− during Acquisition, whereas no SCR differences were detected between CSs during Extinction. Moreover, we analyzed HRV as a quantitative index of the interplay between sympathetic and parasympathetic influences on cardiac activity using frequency‐domain techniques (Akselrod et al., 1981): while the low‐frequency band (0.03–0.15 Hz) is thought to reflect both sympathetic and parasympathetic activity, the high‐frequency band (0.15–0.30 Hz) is considered to be related primarily to cardiac parasympathetic outflows, and thus provides a direct index of vagal activity (Malliani et al., 1991; Pumprla et al., 2002; Task Force, 1996).

This analysis of the spectral components of HRV, used as a noninvasive marker of sympathetic and parasympathetic mechanisms, can dissociate the underlying autonomic mechanisms involved in conditioned and neutral stimuli to fear learning (Schipper et al., 2019). RR‐interval to stimuli were decomposed into their components of heart rate during the Acquisition phase, which allowed us to observe a well‐known triphasic response (Bohlin & Kjellberg, 1979; Castegnetti et al., 2016), consisting of (a) an initial deceleration, (b) followed by acceleration, and (c) a further late deceleration around the time point in which US was expected. Additionally, we were able to expand this window to investigate how cardiac signals converge toward baseline activity after a few seconds, thus revealing a further acceleration followed by a final deceleration.

More importantly for the present purposes, the RR‐interval to CS+ showed larger dynamics than CS−. The cardiac profile was marked by the large continuing deceleration (Figure 1a; D2 slope) in response to the CS+, which was absent in response to the CS−. Since the responses to conditioned and neutral stimuli begin to differ at about 1 s before the expected US onset, the late deceleration component appears to be due to the CS+ presentation rather than US omission, thus reflecting learned anticipation of impending noxious stimulation. During the Extinction phase, CS+ and CS− showed comparable RR‐interval modulation of heart rate. Overall, these findings appear consistent with recent HR data of fear learning in humans (Castegnetti et al., 2016; Paulus et al., 2016; Tzovara et al., 2018).

Crucially, our frequency analysis of HRV adds an important piece of information, revealing transient changes of power spectrum in the high frequency (HF) band, significantly larger after the CS+ than the CS− presentation, peaking approximately around the time point in which participants expected the shock administration (Figures 2a and 3a; Table 1). These HRV components in the range of HF indicate cardiac vagal activation (rather than reduced sympathetic activity) to learned fear, reflecting enhanced sensory intake, and defensive preparedness for the upcoming electrical shock (Appelhans & Luecken, 2006; Bradley, 2009; Schipper et al., 2019). In contrast, HF components were reduced after the presentation of the CS−, consistent with the participant having learned that no shock will follow in this condition. Finally, spectral analyses revealed no significant difference between CS+ and CS− during the Extinction phase (Figures 2c,d and 3b). The fact that the relationships of HRV spectral components to conditioned versus neutral stimuli were similar, irrespective of the quantification method (namely, STFT or point‐process modeling), provides evidence of the reliability of the present pattern of results. Although several earlier studies have argued that the vagus nerve may play a crucial role in fear conditioning, the results of the present study provide the first direct evidence that systematically investigate and, more importantly, quantify its selective involvement in human fear conditioning. For instance, Obrist et al. (1965) reported that fear‐conditioned bradycardia is significantly disrupted when the vagus nerve is pharmacologically blocked (i.e., by administering atropine intravenously). Note, however, that anticholinergic drugs, such as atropine, have both peripheral and central effects, which may deeply affect fear learning and memory in humans and other mammals (Anagnostaras et al., 1999). Therefore, reduction in the decelerative changes of heart rate may stem from impaired fear conditioning, besides vagal blockade at the cardiac level. Moreover, Obrist et al. (1965) failed to demonstrate successful fear learning in the vagal blockade group, since atropine does indeed blocks SCR activity. As such this previous study does not provide definitive evidence for the selective role of the parasympathetic system in conditioned heart‐rate deceleration.

Importantly, the present results may be in line with the neurovisceral integration (NVI) model (Jennings et al., 2015; Thayer et al., 2012; Thayer & Lane, 2000, 2002), which suggests an extensive anatomical overlap between the distributed network of brain areas composing the central autonomic network (CAN), and the neural circuit critically involved in fear conditioning and emotional learning in humans (Maren & Quirk, 2004). Through the sympathetic and parasympathetic––*vagal*––branches of the autonomic nervous system (ANS), the CAN directly regulate heart rate and thus an individual's capacity to generate regulated physiological responses in the context of emotional learning (Ernst, 2017; Schipper et al., 2019; Thayer & Lane, 2000; Thayer & Siegle, 2002). Accordingly, a novel theoretical and anatomical‐functional reinterpretation, namely the neurovisceral integration model of fear (NVI‐f), proposed that the complex interplay between the central and peripheral nervous systems, through a dynamic brain network that extends to the heart, in responding to a fear‐eliciting stimulus (Battaglia & Thayer, 2022). In the NVI‐f model, cascading high‐level cognitive structures, specifically the prefrontal cortex, influence the activity of the amygdala and hippocampus, eliciting neurovisceral fear responses through sympathetic and parasympathetic projections that mediate heart‐related dynamics.

Indeed, phasic HRV enhancement and parasympathetically dominated heart rate deceleration, as observed here when the US is expected, has been associated with emotional regulation (Park & Thayer, 2014), and the ventral portion of the medial prefrontal cortex (mPFC) activation (Roelofs, 2017). This suggests that high‐frequency fluctuations in heart rate may prepare subject to an impending threat, an idea in keeping with our result that a higher concentration of power in the high frequency peaked approximately around the time the US was expected. However, additional studies are required to characterize cardiac dynamics under different experimental manipulations and unambiguously corroborate this result. Beyond fear learning, current findings may prove crucial for the psychophysiological assessment of safety learning (Christianson et al., 2012). This occurs when an otherwise neutral stimulus comes to signal the absence of threat (i.e., Pavlovian conditioned inhibition). The vast majority of safety learning studies have been based on SCRs, which however present complex challenges (Battaglia, 2022; Battaglia et al., 2021; see Laing & Harrison, 2021 for a recent review), notably artifacts of orienting that might mask threat inhibition assessed via SCR. By separating the contribution of the sympathetic and parasympathetic systems, as a means to discern the relative influence of orienting and threat‐related responses, HRV spectral analysis may represent a more reliable and sensitive psychophysiological measure of conditioned inhibition.

A potential limitation of this study is that we did not investigate the CS+/US trials. This is because, in our experiment the ECG signal is artifacted by administering the shock pulse, and moreover, having used a moving‐median approach (Hedges & Shah, 2003) to calculate the RR signal, it is possible that the signal is influenced by receiving the shock pulse, thus producing results that are not temporally reliable. An additional limitation of the study is that we did not monitor and/or control for respiration during fear conditioning. There has been considerable debate on the necessity of controlling for respiration when assessing HF‐HRV during emotional or cognitive tasks (Laborde et al., 2017; Quintana & Heathers, 2014). In fact, respiration has a major influence on the heart rate variability (i.e., the respiratory sinus arrhythmia, RSA), especially in the HF band (Shaffer & Ginsberg, 2017; Yasuma & Hayano, 2004). Although our findings reveal that changes of power in the HF band provide a powerful and robust indicator of fear learning, the current study remains agnostic of the influence of respiration on the RR spectrum during fear conditioning. A recent study of fear learning (Castegnetti et al., 2017), which assessed both respiratory and cardiac activity, reported that respiratory measures are less sensitive than heart beat periods in distinguishing between CS+ and CS−. An intriguing hypothesis is that the vagal response to CS+ may involve a dual effect on HRV: a direct one via the autonomic (e.g., parasympathetic) control of heart rate signal, and an indirect one via the effect of respiration on heart period responses. Future works are therefore required to effectively assess HRV spectra uncontaminated by RSA during fear conditioning. Lastly, we sampled the ECG signal at 200 Hz. The inherent uncertainty of 0.005 s in the IBI interval associated with this sampling frequency did not likely affect the timings discussed in our results, which were presented with a lower precision of 0.01 s. Despite guidelines suggest optimal sampling at 250–500 Hz, or perhaps even higher (1000 Hz), such sampling frequency is necessary when small changes in heart rate variability need to be detected, particularly in pediatric and elderly populations (Singh et al., 2004; Willems et al., 1991), or in patients with pathological decreased variability of RR intervals (Ziemssen et al., 2008). By contrast, large variability of RR intervals was expected in a population of healthy young adults, under our experimental conditions. Indeed, a sampling rate of 125 Hz (Laborde et al., 2017) or 200 Hz (Kuusela, 2013) may behave satisfactorily for both time‐ and frequency‐domain analysis of HRV, and it is a common choice in current literature in young healthy subjects (see Shaffer & Ginsberg, 2017).

Finally, we note that the time‐frequency decomposition of HRV in a laboratory can be interestingly translated into clinical population, to evaluate the abnormal fear learning that characterizes several neurological and psychiatric conditions. Indeed, among individuals with fear‐ and anxiety‐based disorders, HRV tends to be lower at baseline and in response to challenges, reflecting poor inhibition (Kim et al., 2018; Sevenster et al., 2015). Our spectral biomarkers offer the opportunity to have an additional and more precise transient measurement of the sympathetic and parasympathetic nervous systems allowing to quantify the potential difficulties in the modulation of the vagus nerve in response to learned fear in humans.

In conclusion, we were able to characterize the cardiac autonomic dynamics of fear learning in humans, overcoming the difficulties of classical analysis of HRV which failed to disentangle the contribution of the parasympathetic activation and/or sympathetic withdrawal. Crucially, we were also able to reliably trace point‐per‐point transient heart changes in time which constituted a methodological limitation so far. Our findings reveal that spectral HRV analysis can be considered a valuable measure of fear conditioning in humans. Finally, a deeper understanding of the functional interplay between central and autonomic nervous systems could help improve diagnostic protocols, at the same time offering a solid framework for the development of novel and advanced treatments for psychiatric populations, being the “aberrant fear learning” an integral component of many psychiatric conditions (Battaglia & Thayer, 2022).

## AUTHOR CONTRIBUTIONS

**Simone Battaglia:** Conceptualization; data curation; formal analysis; investigation; methodology; project administration; resources; supervision; validation; visualization; writing – original draft; writing – review and editing. **Stefano Orsolini:** Data curation; formal analysis; methodology; resources; software; visualization; writing – original draft; writing – review and editing. **Sara Borgomaneri:** Funding acquisition; resources; writing – review and editing. **Riccardo Barbieri:** Formal analysis; methodology; resources; software; supervision; writing – review and editing. **Stefano Diciotti:** Formal analysis; methodology; project administration; resources; software; supervision; writing – review and editing. **Giuseppe Di Pellegrino:** Conceptualization; funding acquisition; methodology; project administration; resources; supervision; validation; writing – original draft; writing – review and editing.

## FUNDING INFORMATION

This work was supported by RFO grant from the University of Bologna awarded to G.d.P. and by grant from Ministero della Salute, Italy [GR‐2018‐12365733] awarded to S. Borgomaneri

## CONFLICT OF INTEREST

The authors declare no competing interests.

## Supporting information


**FIGURE S1**. Detection of *R*‐waves. The sample‐based envelope detection algorithm applied to an ECG signal (in blue) allows identifying the QRS complex peaks (purple circles). The envelope signal (*y*_env_(*t*)) is represented in yellow, and the amplitude offset of the ECG signal, computed over a moving window of 1 s, is in red. Two detailed views of the envelope are also shown: (1) on the left, the envelope is around the QRS complex peak, where *t*_0_ and *y*_0_ are the time and envelope signal value, respectively, associated with the QRS complex peak; (2) on the right, the envelope signal is at the end of the decay period and tcross is the time at which the envelope equals the ECG signal amplitude and starts updating with the ECG signal until the next QRS complex peak
**FIGURE S2**. RR‐interval. (a) Original RR series (in blue circles), (b) Effect of the linear interpolation (in grey lines), (c) homogeneous resampling to extract the RR interval sequence (in red)

## Data Availability

The raw data that support the findings and the custom codes used to analyze data of this study are available in Open Science Framework at the following online repository https://osf.io/qjyrx/ or further material requests should be addressed to Dr. Simone Battaglia.
