# Supplementary material for: Characterizing cardiac autonomic dynamics of fear learning in humans
Source: Psychophysiology. 2022 Jun 7;59(12):e14122. doi: 10.1111/psyp.14122 (PMC9787647; doi:10.1111/psyp.14122)
Supplement: Supplementary file 1 — FIGURE S1. Detection of R‐waves. The sample‐based envelope detection algorithm applied to an ECG signal (in blue) allows identifying the QRS complex peaks (purple circles). The envelope signal (y env(t)) is represented in yellow, and the amplitude offset of the ECG signal, computed over a moving window of 1 s, is in red. Two detailed views of the envelope are also shown: (1) on the left, the envelope is around the QRS complex peak, where t 0 and y 0 are the time and envelope signal value, respectively, associated with the QRS complex peak; (2) on the right, the envelope signal is at the end of the decay period and tcross is the time at which the envelope equals the ECG signal amplitude and starts updating with the ECG signal until the next QRS complex peak FIGURE S2. RR‐interval. (a) Original RR series (in blue circles), (b) Effect of the linear interpolation (in grey lines), (c) homogeneous resampling to extract the RR interval sequence (in red) [file PSYP-59-e14122-s001.docx]

**SUPPLEMENTARY MATERIALS**

**Detection of R-waves**

The algorithm for identifying QRS complex peaks was initially developed for a real-time application in pulsimetry and subsequently applied successfully to recorded ECG data from this study. The algorithm is based on the concept of envelope detection (Figure S1).

**
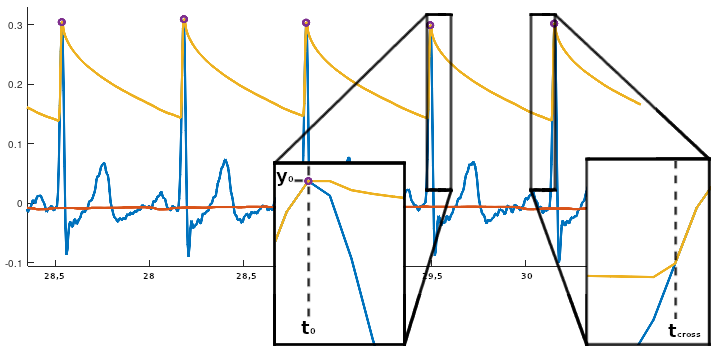
**

**Figure S1. Detection of R-waves.** The sample-based envelope detection algorithm applied to an ECG signal (in blue) allows identifying the QRS complex peaks (purple circles). The envelope signal (*y*_env_(*t*)) is represented in yellow, and the amplitude offset of the ECG signal, computed over a moving window of 1 s, is in red. Two detailed views of the envelope are also shown: (1) on the left, the envelope is around the QRS complex peak, where *t*_0_ and *y*_0_ are the time and envelope signal value, respectively, associated with the QRS complex peak; (2) on the right, the envelope signal is at the end of the decay period and tcross is the time at which the envelope equals the ECG signal amplitude and starts updating with the ECG signal until the next QRS complex peak.

The value of the envelope signal (y_env_(t)) is initialized with the value of the ECG signal amplitude ECG(t). When t increases and a relative maximum is reached, the amplitude at the maximum is stored in y_0_ and the time at the maximum in t_0_ (see Figure S1 – detailed view on the left). Subsequently, at increasing t, a decay of y_env_(t) starts by updating y_env_(t) using the formula

$$y_{env}(t)=y_{o}- d\cdot| y_{0}- y_{offset}(t)|$$

where y_offset_ (t) is the offset of the ECG signal computed over a moving window of 1 s. y_offset_ is removed from y_0_ to avoid numerical divergence, and the absolute value of such a difference is multiplied by a decay function d

$$d=r\cdot\sqrt{t-t_{0}}$$

which employs the square root of the time passed from t_0_ in seconds weighted by the decay rate (r) set at 0.65. The decay of the envelope over time ends when y_env_(t) equals the value of the ECG signal amplitude (at t_cross_ time, see Figure S1 – detailed view on the right). From that time, y_env_(t) is updated again with the value of the ECG signal amplitude ECG(t) until the next detection of a QRS complex peak. The envelope is then necessary to distinguish the QRS complex peak from other relative maxima (e.g., the T wave peak) using defined amplitude and time ranges. In particular, a peak is recognized as a QRS complex at time t_0_ if the detected amplitude in the ECG signal (y_0_) lies within a tolerance (p = 80%) of the envelope (i.e., p*y_env_(t_0_)< y_0_< y_env_(t_0_)). Finally, to avoid spurious detections of relative maxima in the QRS complex, the time from the previous t_0_ must be greater than 0.45 s. This last constraint sets a boundary to the maximum detectable heart rate of 134 beats per minute.

**RR-interval sequence**

The RR-interval sequence was obtained by homogeneous resampling after interpolating the IBI sequence at 10 Hz. It is important to highlight that a frequency between 4 and 10 Hz is considered an appropriate resampling rate for the study of autonomic regulation since it enables to compute of reliable spectral estimates. All IBI's are used as depicted in Figure S2, in which the RR series is shown (in pane a), then linear interpolation is applied (pane b), and finally, a homogeneous resampling has been used to extract the RR interval sequence (red signal in pane c).


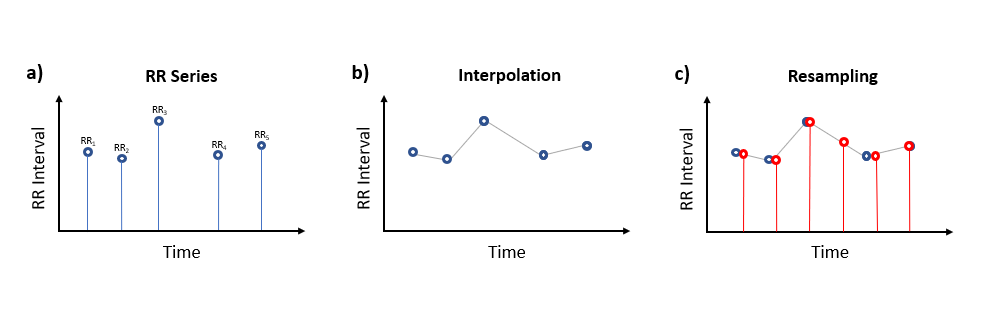
 **Figure S2**. **RR-interval.** a) Original RR series (in blue circles), b) Effect of the linear interpolation (in grey lines), c) homogeneous resampling to extract the RR interval sequence (in red).
